# The Army Surgeon—An Address to the Graduates of the Medical Department of the University of Buffalo, February 24th, 1863

**Published:** 1863-03

**Authors:** Thomas F. Rochester

**Affiliations:** Professor of the Principles and Practice of Medicine


BUFFALO

### Medical and Surgical Journal

VOL. II. MARCH, 1863. NO. 8.

### ORIGINAL COMMUNICATIONS.

ART. I.—*The Army Surgeon—An Address to the Graduates of the Medical Department of the University of Buffalo, February 24th,* 1863, *by* THOMAS F. ROCHESTER, M. D., *Professor of the Principles and Practice of Medicine.*

BUFFALO, Feb. 25, 1863.

DR. J. F. MINER:

Dear Sir—As you, and several of my professional friends, have expressed a desire that the following address should be published in the Buffalo Medical Journal; it is at your service. While my thanks are returned for the compliment, justice to myself and to the subject, requires that I should add, that the address was hurriedly thrown together, within the last ten days, in such time as I could snatch, from extreme professional occupation.

Yours, very truly,

THOS. F. ROCHESTER.

It rarely happens, on an occasion like the present, a quasi-professional epoch of annual recurrence, that a theme, suitable for the customary charge or address to the graduates in medicine, should be afforded by any topic of general and popular interest; such, *unfortuuately,* is not the case, in this sad year of war and wicked rebellion, A. D., 1863.

In selecting "The Army Surgeon" as the title of his discourse for this evening, it has seemed to the writer that the subject pertains to the whole audience, directly or remotely, as well as to that small portion of it comprised by those who have just received the Academic honors, to which it is hoped they are fully and honorably entitled.

When, on the 12th of April, 1861, the lightnings were discharged from the ominous war cloud that had so long darkened the southern horizon, when the reverberations of beleaguered Sumpter's guns woke a responsive echo in every patriot's breast; when the great heart of a wounded and insulted nation—wounded by a most foul and parricidal blow, pulsated with a thrill that vibrated from the shores of each ocean barrier, and extended to the utmost limits of the loyal north; when the proud, strong Mother, America, uttered her cry of sorrow, anguish and anger, how eagerly and earnestly did her sons answer her appeal for succor, and none more promptly than those who had made medicine their profession. What their position, and what their labor and duties, let us now endeavor to describe. According to the Army Register, the grade of the Medical Corps is as follows:—Surgeon General, with the rank of Brigadier General—Assistant Surgeon General, with the rank of Colonel,—Medical Inspector General, with the rank of Colonel,—Medical Inspector, with the rank of Lieut, Colonel,—Surgeon, with the rank of Major,—Assistant Surgeon, with the rank of Captain,—Assistant Surgeon, with the rank of 1st Lieut.,—Surgeon of Volunteers, with the rank of Major. The salary of the Surgeon-General is $2,740 per annum; that of the others gradually falls to $1,500. These sums include the allowance made for rations, horse keeping, servants' wages, et cet. It will be seen that the remuneration is less than that of other officers of the same grade; it is not much more than sufficient to pay necessary expenses. The duties of the first four (in grade,) are administrative and supervisory, and involve an immense amount of labor, talent and discretion; but as these services are, to a great extent, bureaucratic, and thus foreign to the illustrative purpose we have in view, while we pay tribute to their worth and indispensability, let us pass to the consideration of those who are in direct personal relation with the rank and file of the army, viz: the hospital, field and regimental Surgeons. Of these, there are about 2200 in the service, besides some 1500 Contract Physicians, engaged by the month for specific duties; a brigade in itself, and yet, in point of numbers, by the last report of Surgeon-General Hammond, entirely inadequate to the work in hand. Through his efforts, a bill was recently introduced into Congress, to increase this force, but through most mistaken notions of economy, failed to pass. "Instead" says the American Medical Times, "of meeting the questions which the measure suggests, fairly and candidly, SENATORS played upon each others sympathies, by relating incidents of the cruelty of medical men to sick soldiers, of their incompetency, and even of their knavery. Not a Surgeon was spoken of in a complimentary manner; of the large number who have been killed, while caring for the wounded on the field of battle, there was not a breath of praise," and, let us add to this comment upon legislative action, that, while we admit that in so large a number of men, there must, from mere human infirmity, be some fools and some knaves, as a body, the medical corps may safely challenge comparison with any department of the army. Every full regiment is entitled to a Surgeon and two Assistant Surgeons, the second Assistant having been recently allowed as the necessary labors have been found to be more arduous than anticipated, and also to the intent that there should always be two efficient Surgeons, should sickness or casualty befal one medical attendant. The duties of these officers commence with the organization of the regiment; they should, in fact, be preliminary to the enrolment of men enlisted by the Recruiting Officer. Every applicant should be thoroughly examined, and if any of the many infirmities are detected, that *technically* constitute "physical disability," those thus disqualified should be rejected at once. It is true that the examination required by law, is generally formally made by some medical man, appointed by the Government for that purpose, (reference is here made exclusively to Volunteers,) but this duty would be much better performed by the Surgeon of the regiment. Their *amour propre*, respecting the efficiency of their *own* regiment, and the prospective lightening of their necessary professional labors, to say nothing of duty and patriotism, would cause them to be careful and particular in their investigations. One well meant error has often been committed,—the enlistment of boys. It is no longer venial, for we have learned from sad experience, the justice of Napoleon's indignant remonstrance with the Legislature of France; "Shame on you! I demanded a levy of three hundred thousand *men*, but I must have *grown* men. Boys serve only to fill the hospitals and encumber the road sides."

But there are abuses that are little dreamed of in the formation of volunteer regiments, pardon the digression while reference is made to them, in hopes that the exposure may prevent, in some measure, their continuance. Recruiting officers, whose commissions are dependent upon their enlisting a certain number of men, are little scrupulous as to the quality of the material they get together, and noble and patriotic and self-sacrificing as are the motives of a majority of our citizen soldiery, there are many, very many, who enter the hallowed ranks of the champions of Union and Liberty, only to pollute and weaken them. Impelled by destitution, by greed of possible lust and rapine, by large bounty, by fear of draft, by commutation of sentence of imprisonment, hundreds of wretches, some half blind, some deaf, some maimed, some fearfully and incurably diseased, have contrived to pass the medical examiner. They are counted, on the one side, as "good enough to stop a bullet," on the other, (*i. e.* by themselves,) as having no such intention; they never mean to be brought under fire. When they have secured their bounty, they desert, or they go into the hospital and stay in it, or they obtain sick leave and prolong it, indefinitely, still sporting their buttons, or they visit Washington, and when they have staid there long enough, their physical disability is made apparent. and they are sent home, at the expense of the government, to prolong their impostures, exciting the commiseration of many, as patriotic soldiers, worn out by the toils and privations of war. If forced into action, these are the skulkers, tbe panicists, the stragglers,— Sad as is the avowal, these are no random assertions; the speaker is personally cognisant of blear eyed, of deaf, of lame, of consumptive, of epileptic, and of otherwise diseased persons, who have been admitted into volunteer regiments, either through their own falsehood, or through the aid and connivance of would-be-officers, who, when remonstrated with, replied, "oh, we keep these fellows until we perfect our organization," *i. e.* our commissions, "they will never be mustered in"--but they contrive to be, most of them, and are only mustered *out*, when a fight is imminent. In view of these facts it easy to understand the deterioration, the demoralisation, and the decimation of which we hear so much. "It can scarcely be credited, and yet it is stated, on the best authority, that a single Medical Inspector in New York City has regularly passed two hundred and fifty men, daily, occupying eight hours each day. The examination of each recruit in this case occupied, on an average, less than *two minutes*," (*American Medical Times*, August 30, 1862.)—15 cents each, inspection fee, $37.50 per diem. The inference is obvious. Were this inspection duty in the hands of the proper surgeons, each for their own regiment, the evil could not attain any magnitude, but the medical officers, usually the very last appointees, take the rank and file as they find them. The newly formed regiment is at its rendezvous in camp or in barracks. At first the medical care is chiefly hygienic—the food is examined as to quantity, quality, and cookery; water as to sufficiency and purity; sleeping and living apartments as to neatness, ventilation, sunlight and miasmatic exposure, dampness, and crowding; clothing as to protection, warmth, and durability; vaccination and re-vaccination, to secure the inestimable boon of Jenner; proper cloacæ for the reception of the waste and debris, engendered of necessity wherever human beings are long congregated together; the selection and arrangement of suitable hospital accommodations for all who may be ill or injured; and the securing and preparation of proper medical and surgical stores and supplies. Every morning, at a special hour, "sick call" is held, when the orderly sergeant of each company hands in a list of all persons either ill or claiming to be so, those who are able, accompanying him to the surgeon's quarters, where they are prescribed for, the hospital steward dispensing medicines as directed. Those too ill to present themselves, are visited and sent to the hospital or hospital tent—this latter is 14 feet long, 15 feet wide, 11 feet high in the centre, and 4½ feet high at the sides. It is so constructed that it may be enlarged in length to any extent desired; each hospital tent will accommodate comfortably 8 or 10 patients; three of these and one "Sibley" and one common tent are allotted to each regiment for the use of the sick and their attendants. "Sick call" does not preclude all requisite attention at other hours. The surgeons find it necessary to be extremely particular in their examinations, as men often feign various diseases very adroitly, in order to escape drill, picket duty, and other required service. Too much importance cannot be attached to the location of camps and barracks. The responsibility of the selection of place should rest with the Medical Director, or in his absence with the regimental surgeon. For want of proper surveillance in this respect, an infinity of disease is often engendered, of which one instance, leading to terribly disastrous results, has recently come to the knowledge of the speaker. The 161st Regiment N.Y. Volunteers, composed of able bodied men, mostly farmers' sons were ordered to rendezvous at Elmira, preparatory to being mustered into service. They were placed in barracks, shockingly located, that had been repeatedly occupied, that were not suitably ventilated, and had not been properly cleaned and purified. The walls and the floors were recking with the typh poison of animal and vegetable effluvia. Like a great vampyre trap, it was thirsting for victims; and they came. Soon these stalwart sons of the soil, types of manhood, began to sicken, a low fever, a blood poison commenced its fatal work. The regiment had met a foe more deadly than the bullet, it was more than decimated. So general and so terrible was the pestilence, that to avoid annihilation, the regiment was disbanded, the men were scattered, but the fever fiends went with them to their homes and seized them there, and not only them, but their friends and attendants. The lesson is a severe one; let us hope it was not lost.

It has been stated above that the responsibility of selection of place should rest with the surgeon; this, however, is not sufficiently recognized for it often happens that that officer is not even consulted respecting location. "At the home camp or barracks," says Dr. Hunt, "the surgeon is nobody, but away on the march, after a week of camping and bivouacing, he is the real Colonel of the regiment." Making due allowance for this figure of speech, the idea inculcated, is the importance of the position, and the necessity of its being properly filled; and this leads us to say a word as to the appointment of medical officers. In New York, and in most of the other States, the Governor assigns them to their respective regiments, on the certificate of a Board of Medical Examiners, to the effect that they are duly qualified, having passed the requisite examination. This is true in the main, and if strictly adhered to would be the wisest and safest course, but there are some exceptions. A noted politician and a notorious doctor, ambitious of military honors, visited the State capital, ostensibly to be examined, the form even was omitted. "Your reputation has preceded you," said the polite official. Here is your commission." After a year of incompetency he resigned, and returned a debased and degraded sot. A surgeon, a *strong* man, who had undergone triumphantly a rigid examination, had sought the post in vain. This was perhaps a solitary instance, but there is another well intended error, frequently committed. A regiment is formed; there are many aspirants for the surgeoncy. Some of them from social rather than professional qualifications, receive, in caucus, a majority of the officers' votes. The State authorities are advised of the fact, the *elected* ones pay them a visit, with proper credentials, i. e. with commendatory letters from the officers, and from personal friends, given with good intent, but on insufficient grounds, and whatever the result of the medical inquiry, they invariably return with their commissions. Sad mistakes have occurred in this way, and have been sorely visited upon the injudicious officers who have used their influence to secure the appointment of A. B, or C. This is the source of the announcement, "discharged for incompetency," of which there were no less than twelve in the last fortnight of January, 1863. But let no one suppose that these areaug ht but exceptions. Generally the medical staff of the volunteer army is composed of good men and true, of men who go not for place or for gain, but from patriotic and philanthropic motives, who abandon the comforts and endearments of home, and not unfrequently a practice, the emolument arising from which is double or treble the salary they are to receive.

Let us follow these officers of war, whose mission is to save, not to destroy life; whose efforts extend equally to friend and foe, and who combat only against the diseases of camp and the carnage of battle. On the transport and on the railway car, it is their duty to see that there is proper space and ventilation, to warn against excesses of food and drink, and to attend to accident or illness as they may occur. On the march they note all stragglers, all who fall out from real or feigned fatigue or sickness, discriminating between them, giving a written permit or pass, to the one for the following ambulance train, and ordering the other to his place in the ranks They report to the proper officer the effect of the weather, of the nature of the country, of the length and rapidity of the march, as to its influence upon the health and endurance of the troops, and are always expected to note and report any condition or circumstance that may in any way prove detrimental to the physique of the army. When a halt is made for the night, the surgeon points out the most appropriate ground to be occupied, especially avoiding marshy or malarious exposure. When the troops bivouac, i. e. lay on their arms, at night, before the foe, the same precautions, are as far as possible, observed. When camp is made more or less permanently, either upon friendly or hostile soil, the surgeons have the same hygienic measures to enforce mentioned as necessary to the primary or home encampment; especially do they have to insist upon sufficient bathing of the person and change of undergarments, and to look to the daily opening and ventilation of the tents. Neatness, air and sunshine being wondrous antiseptic agents. It does not do to pitch a tent for more than a single night on a growing wheat, oat or other cereal field, as fevers, diarrhœas and dysentery emanate from these green couches of nature.— Care must be taken also that straw employed for bedding is neither damp or mouldy, and that however dry and bright, that it is frequently turned and shaken, as it is thought from parasitic or fungoid growths, to originate a disease akin to if not identical with measles. The great prevalence and mortality of rubeolus disease in both armies, at the beginning of war, has, with some show of reason, been attributed to the general and unaccustomed use of straw.

The hospital tent now begins to find its uses, and the Surgeons ample occupation; they have to study the condition of soil, water and climate, and the process, so called, of acclimation. They have to determine who are fit for full. and who for partial duty; and who from temporary indisposition, should, for the time, be excused from long drills and from the exposures of picket and scouting service. A large amount of writing has always to be done; daily and weekly reports are made, stating not only the number of sick, and their diseases, but also the number and general sanitary condition of those accounted well. These reports are sent to the medical inspector, and by him embodied with others to the Surgeon-General. After a battle, this clerkly labor is immense, as a full and minute statement of all casualities is required. There are always to be made out or signed, applications for leave or furloughs, or for discharge from causes connected with injury or ill health, requiring, absolutely, official examination and certification. Then there are the requisitions for stores and supplies, and the acknowledgment of the receipt of the same. Besides all this, especially with Surgeons of Volunteers, there is a great amount of correspondence with the relatives of sick soldiers; they are appealed to as possessing the only reliable knowledge, and should never, if possible, withold the desired information. It rests mostly with them, also, to give notice to friends at home, of the severe illness or death of those under their professional charge. It seems proper here to allude to an illness peculiar to *absentees.* It is called Nostalgia, or Home sickness, and effects especially the younger soldiers, the lads of 15 to 20, who have left, for the first time, their tender homes, to engage in the dread struggle that demands every attribute of manhood and self-reliance. The disease manifests itself by great depression of spirits, by extreme nervousness, by inaptitude for exertion, and is soon followed by loss of appetite, and derangement of the digestive functions. It is always aggravated by interruption of communication with home, by losses and disasters, and especially by repeated retreats. If not relieved, it terminates in insanity or death. The Surgeon cannot be too gentle with such patients, he must breathe to them words of hope and encouragement, he must secure for them, if possible, relaxation and exemption from the more arduous duties, for they are neither shamming or cowardly, and if a fight is imminent, they will rush to the ranks, and in the contest prove their valor with the best; and now that this allusion has led us to it, let us examine the Surgeons' duties and position on the battle ground. Notice having been given of the coming engagement, the medical directors select certain contiguous buildings, or sheltered places, with temporary structures, as hospitals for the reception of the wounded. These are assigned to the requisite number of surgeons, and it is their business to see that everything that can possibly be required is in readiness. (At the first Bull Run, water, sponges and lights were in very insufficient quantity.) The regimental surgeon, his regiment being drawn up in line of battle, selects a position as near as possible, secures such shelter as is practicable, including, if within his power, spring or running water and shade, prepares his operating table, generally made of camp stools or barrels and two broad boards, plants his red hospital flag, and displays his green sash, as the official badge of his quasi neutral position, The assistant surgeon, with stretcher bearers, and other detailed men, follows his regiment about one hundred yards in the rear; an orderly, carrying a knapsack, containing instruments, bandages, stimulants, et cet., accompanying him. The medical directors and medical inspectors are stationed here and there, to see that their orders are fully carried out,—that the ambulances are properly stationed and supplied, ready to be brought up whenever and wherever uecessary, and, in short, to see that no means are wanting to mitigate the sufferings of the injured. These are the preparations, and how much depends upon their efficiency is needless to insist. There is a moment's stillness, 'tis the hush of the tempest; yon flashes a light; 'tis the gleam of a meteor of death. The quiet is broken. The heavy thud of artillery, the rattling of musketry, the screaming of shells, the shouts and the voices, and the commands of men, the trumpet's brazen note, the bugle's call, and the martial music of drum and fife, are all strangely distinct, yet mingled, and over all, as if to veil these scenes of blood and strife, there rises a dim canopy of dust and sulphurous smoke, and under its shade, here and there, lie prostrate the forms of many, a moment before eager and erect, and now, with these sad evidences of mortal combat before him and around him, the surgeon seeks to lessen its horrors. As each soldier is wounded, he passes to the rear, if able, when not, he is carried thither by the ambulance corps, or by two of his comrades, when so ordered by the proper officer, and not otherwise; "for when this is left with the men" says the Duke of Wellington, "in an hour a whole battalion will tail off after some fifty wounded." The surgeon or assistant on duty, directs those slightly wounded to the regimental hospital position, causes those severely injured to be transported thither, and whenever severe hæmorrhage is going on, uses, on the spot, the appliances for arresting it. Those so seriously wounded, that it is manifest they can live but a short time, are tenderly cared for, but are rarely removed, especially when there is pressing necessity to look after those who yet have a chance of life. Operations other than those for staunching the flow of blood, are rarely made literally upon the field. This was not the case however with the gallant Heintzelman, who, while sitting in the saddle, had a bullet extracted from his arm by surgeon Hooker, and then for hours strove to redeem the failing fortune of the day.

An impression prevails that the surgeon is not exposed to the perils of the battle ground. This is a mistake. Staff surgeons remain with their Generals, and are in as much danger as any of the officers. The surgeons who follow closely in the rear of their regiments, are often thrown, by change of position, directly in the line of fire; three were killed outright at the battle of Antietam. The surgeon of the 6th N. Y. Cavalry, in a letter to the writer says: "I have often been exposed to fire in the discharge of my legitimate duties, for I never had any desire to seek unnecessary danger." The red flag of the field hospital is usually respected, not always however in the madness and rage of strife, and in a spot so near the scene of contest, chance shots must occasionally fall. At this, the post of the senior regimental surgeon, operations are made upon those who cannot safely be trusted to the delay and fatigue of further transportation; here shattered limbs are comfortably arranged with temporary dressing; the ambulances are properly filled, and stimulants and anodynes are given to such as require them; here ready wit and mechanical ingenuity are always called into requisition, The store of the usual appliances is often exhausted, and their place must in some way be supplied. An unsupported fractured limb will cause great suffering and possibly death; barrel staves, bands of straw, and even bayonet sheathes, have been admirably employed as temporary splints. A question of precedence as to the surgeon's services sometimes arises. It is a rule of usage, that when officers and privates are equally injured, to first attend to the former, but where a private has the most serious wound, the officer must give way; but disputes on this point seldom arise. Soldiers of all grades manifest great heroism and self-abnegation, voluntarily urging that assistance be first given to those who most need it.

The strife is over; should the day be lost, what is the duty of the surgeon? It is his privilege to retire, he may be ordered to join in the retreat, and if so, should obey, but it is also his privilege to remain with his poor suffering companions, and what man with a human heart would hesitate which to do, At the commencement of this sad war, this was no common sacrifice, for it entailed hardship, imprisonment and threatened retaliatory death, as in the case of Dr. Slocum, Assistant Surgeon in the Navy, and two years ago a graduate of this College. Yet scores who might have saved themselves without dishonor, refused to to leave *their wounded*, and from this magnanimity of the federal surgeons arose the subsequent agreement "that surgeons who remained with those requiring their aid, should not be held as prisoners of war." Should the day be won, or should the battle be drawn, the surgeon must continue his labors. The dread field is explored by parties detailed for the purpose, and through successive days and nights must he prolong the almost unremitting toil. It is unnecessary to more than allude to the great permanent hospitals, more or less remote, to which ultimately the sufferers are removed, but there are two features in connection with them, that must not be passed over. *First*, there are *women* there, the women of America, not hireling nurses, but delicate, fair, sisterly, motherly women, patriots, philanthropists, Florence Nightingales, pilgrims to a shrine more holy than that of Mecca—kind words, cheerful smiles, the light hand that soothes the aching brow, the sympathising heart that listens to the tale of home, that receives the messages, that writes letters, the gentle step that reveals the feminine presence—these are their true *materia medica*, and more potent, often, then all the drugs and appliances of man's rude contrivance. Then are they indeed the "ministering angels" of whom Byron sung so sweetly, mingling albeit the bitter with the sweet.

*Secondly*, there is the powerful aid of the Sanitary Commission; and not only in the hospitals and camps, but on the battle field itself, is this philanthropic association ever foremost in his herculean labors. At Seven Pines, Antietam, Murfreesboro, everywhere, where the battle storm has raged, physicians, nurses, supplies, transportation, and even food, all best of their class, have, through wise forecast and provision, been close at hand and pressed forward, days in advance of the Government trains. How much suffering has been spared, and how many lives have been saved by this most noble private enterprise, it is almost impossible to conceive, much less to narrate. Let us by voluntary aid assosiations, and in every possible manner, lend a helping hand to this efficient charity.

We have thus briefly sketched some of the labors of the medical corps. As to its general merit, there can be no question, but it is always gratifying to have the corroborative testimony of disinterested persons, and therefore the following foreign statement is quoted: "The Medical Director of the British Army has expressed the most decided admiration of the United States Army Medical Department. He obtains direct information from an agent of his own department, Inspector-General Muir, who is now on a tour of medical observation in our army. General Muir was present at the battle of Antietam, and remarked that no battle field was ever as quickly and abundantly provided with every necessary for the wounded." Of this medical corps what are its rewards and its stimulants to exertion?—nought, save the proud consciousness of doing good, and of contributing to humanity and to the profession the knowledge gained by a wide and diversified experience; for there is no promotion, no higher rank to look to, and yet, there *are* exceptions to this almost universal rule. At the battle of Palo Alto, twice had a destructive battery been attacked, and twice had the assaulting party been repelled with loss; a third charge was directed, but the men faltered, most of their line officers had been killed or wounded. A young man with M. S. (Medical Staff) upon his shoulder straps, stepped up to the commanding officer, earnestly entreated him, and at length obtained his assent. Dashing to the front, his eyes sparkling, and cap raised on uplifted sword, he cried: "Come boys, he says *I* may lead you." The effect of his enthusiasm was electric; with a wild shout they rushed forward, and this time carried the battery. Their leader was Assistant Surgeon Brown, afterwards the gallant but ill-starred Col. Brown of our glorious 100th Regiment. Peace to his ashes 'neath mournful seven oaks! For this brave deed he received a captain's commission. Strictly, he should not have been allowed to go, but success makes many a fault venial, and this was one like Alvarado Hunter's happily eventuated. At the siege of Sumter, Surgeon Crawford was assigned to the charge of a gun, and for his good conduct was appointed a Major of infantry. Assistant Surgeon Myer, our townsman, is now the head of the signal corps, the creation of his inventive genius, and has the rank and title ofMajor.

As in civil practice, so in war, the result of the surgeon's labors is not limited to himself or his own era. The extended and diversified experience of a great campaign, always teaches many useful lessons of disease, its course and treatment and of operations new and variously modified. This very war has proved that many an injured arm, that five years ago would have been condemned to amputation, may be saved by the new process of resection and exsection of bone, as just exemplified in the wound of the brave General Brown, who repulsed the rebels four-fold in strength, in their last attack on Springfield. The Crimean war established the efficacy and comparative safety of the anæsthetic agents, chloroform and ether.

War has given to the world many great lights of medicine, as noble and pious old Ambrose Paré, who, on being complimented for his success, gave a lesson to us all, in his modest and earnest reply, "I dressed the wounds; God healed them;" as Hennen, Percy, Guthrie, Larrey, Armond, Richter, Mann and others. May we not have a galaxy of names of American

Military Surgeons as bright as these? The sad occasion is not wanting. May God in his mercy cut it short. May smiling Peace, with Union restored, and Liberty a *reality* to every human being under the folds of the "Star Spangled Banner," again brighten our land now darkened by the shadow of mourning habiliments, that cover so many desolate hearts within the broad limits that extend from Maine to Texas. But if it is His will, that the flames of the fiery furnace of affliction and purification, foredoomed it would seem to every nation, should continue to rage, let us take comfort in the thought, that the mission of *our* profession in the struggle is one of mercy. The University of Buffalo is well represented in her sons in this respect. The two classes preceding your own have furnished at least twelve medical recruits, and how well they have borne themselves is attested by the fact, that where hundreds of applicants have been rejected by the strict examining boards of the regular army and navy, not one hailing from this University is of the number. Two even of your own class,* whose names you recognise in this evening's list, are now the honored recipients of their professional title in their Country's service, in anticipation of their Academic degree. One there was, an alumnus of older date, a student of one of my colleagues, known to some of you, known and loved long and tenderly by many of you; (†) known and respected for his manly qualities and professional skill, throughout this great city; one there *was*, alas! that it should be so, the Surgeon of our 21st regiment, who has gone to his last home, and who, "good and faithful servant," if ever human being deserved that title, has entered upon his reward. When he accepted the position, every parent, every brother, every sister, every one interested in those who composed this, our first regiment, experienced a sense of satisfaction, confidence and relief, and well was their trust fulfilled. From first to last he more than did his duty; while he was with it, through his precautionary efforts, the regiment was singularly exempt from the sickness that desolated others. So general and well established was the reputation of this "model army surgeon," that his assistance and counsel were constantly sought by the medical attendants of many of the other regiments. He was soon promoted to the position of Brigade Surgeon, and for a time served in that capacity, but would never abandon his regimental position, for said he, "I promised to take care of the boys, and I will never desert them." Dis-

* Heman Potter Babcock, Assistant Surgeon, U. S. N.; Fletcher M. Follett, Assistant Surgeon, U. S. N.

† The Audience.

charging fully his proper offices, he often extended them; he would visit the exposed pickets, not to see if the men were doing their duty, but to see if they were able to endure and to perform this hazardous service. To many a weary soldier did he send relief, and many a youthful and delicate lad, was, through his interposition, excused from this perilous watch. On the march, he was frequently seen to dismount, and placing a worn and foot-sore comrade in the saddle, would shoulder his musket and heavy knapsack, and thus burdened, walk miles in the Potomac mud. During a battle, on one occasion, he walked cooly into the line of fire, and stood there as calmly as if no danger were near. This was done, not from bravado, for than this, nothing was more repugnant to his nature, but probably he saw some signs of faltering, and sought by his mere presence to encourage and reassure the men. At last came the sacrifice. The fierce battle of Antietam was fought and won. Five days and nights of incessant toil and anxiety followed. The work of at least three men was imposed upon him, and was accomplished, and then, the physical frame that had beed upheld by the indomitable will, and the philanthropic soul; the physical frame, long before shattered by the deadly marsh poison, yielded, and he came back to his Lake Erie home, *to die.* His death was more than a loss, it was a calamity. Attest this! bereaved regiment, deprived of guardian as well as physician. Attest this! citizens of Buffalo, by the aching void that your hearts experience. Attest this! medical men of Erie County, by the cherished remembrance of one, whose honor, probity and rectitude will ever sanctify and endear his memory. It is customary, gentlemen, to point an exemplar to those embarking upon the path of professional life. He of whom we have just spoken, possessed, when in your position, no advantages of place or of education to which you may not lay claim. When the inevitable end, which closes every mortal career, comes to you and to us, may it be gilded with as pure a radiance as that which hallows the name of CHARLES H. WILCOX.

